# Hospital-Based Health Technology Assessment of Machine Perfusion Systems for Human Liver Transplantation

**DOI:** 10.3389/ti.2022.10405

**Published:** 2022-05-27

**Authors:** Paolo De Simone, Davide Ghinolfi

**Affiliations:** ^1^ Hepatobiliary Surgery and Liver Transplantation, University of Pisa Medical School Hospital, Pisa, Italy; ^2^ Department of Surgical, Medical, Biomolecular Pathology and Intensive Care Unit, University of Pisa, Pisa, Italy

**Keywords:** liver transplantation, machine perfusion, health technology, health technology assessment, hospital, patients

## Abstract

Based on published data, we have carried out a hospital-based health technology assessment of machine perfusion in adult liver transplantation using cold storage as a comparator, and within the perspective of a national health system-based hospital practice and disease-related group reimbursement policy. A systematic literature review on machine perfusion for adult liver transplantation was conducted exploring the Pubmed, CINAHL, Scopus, Embase, and Cochrane databases. The literature was analyzed with the intent to provide information on 6 dimensions and 19 items of the hospital-based health technology assessment framework derived from previous studies. Out of 705 references, 47 (6.7%) were retained for current analysis. Use of machine perfusion was associated with advantages over cold storage, i.e., a 10%–50% reduced risk for early allograft dysfunction, 7%–15% less ischemia reperfusion injury; 7%–50% fewer ischemic biliary complications, comparable or improved 1-year graft and patient survival, and up to a 50% lower graft discard rate. Hospital stay was not longer, and technical failures were anecdotal. Information on costs of machine perfusion is limited, but this technology is projected to increase hospital costs while cost-effectiveness analysis requires data over the transplant patient lifetime. No hospital-based health technology assessment study on machine perfusion in liver transplantation was previously conducted. From the hospital perspective, there is evidence of the clinical advantages of this novel technology, but strategies to counterbalance the increased costs of liver transplantation are urgently needed. Further studies should focus on the ethical, social, and organizational issues related to machine perfusion.

## Introduction

Health technology assessment (HTA) is a research-based, practice-oriented assessment of available knowledge on both the direct and intended consequences of health technologies (HT), and on their indirect and unintended consequences, in the short and long term (1). The consequences include clinical benefits (i.e., efficacy, effectiveness) and the economic and organizational impact (efficiency), as well as the social, ethical, and legal implications associated with the HT being assessed (1) (Table 1).

**TABLE 1:** The scope, aims, and perspectives of HB-HTA.

Domains	Definition
Scope	• Provide hospital decision‐makers with information on the effects and implications of introducing a new HT into the hospital
Pre-requisites	• Information on HT has to be relevant, comprehensive, objective, and reliable
	• It has to be specific to the context of the hospital where the HT of interest is to be introduced
Aims	• Take better-informed decisions supporting effective health practices
	• Facilitate more efficient investment decisions
	• Allow hospitals to save money by reducing unnecessary use or avoiding inappropriate investments
	• Facilitate best clinical practices
	• Improve patient safety
	• Engage key opinion leaders in decision-making processes
	• Inform stakeholders on the rationale of managerial decisions and resource investments
Perspectives^a^	• Hospital managers
	• Policy makers
	• Healthcare payers
	• Key opinion leaders
	• Hospital healthcare staff
	• Patients and their families
	• Community
	• Stakeholders
	• Scientists, researchers
	• Industry

Note. HT, health technology; HB-HTA, hospital-based HTA; HTA, health technology assessment.

a In HB-HTA reports, the pre-eminent perspective is that of hospital managers. However, due to the multidisciplinary character of any HTA process, all of the indicated perspectives are to be considered.

Science‐based information is of special importance for hospitals as they are the entry point for new technologies. Over the last decade, the practice of liver transplantation (LT) has witnessed the introduction of *ex-vivo* machine perfusion (MP) systems for both donation after brain (DBD) and circulatory death (DCD) (2). This emerging technology has the potential to improve the outcome of LT, especially when extended criteria donors (ECD) are used (2). However, post-marketing HTA of MP is still inadequate as per the standards of national HTA agencies, and to the best of our knowledge no assessment of MP from an HTA agency has ever been performed.

Hospital-based HTA (HB-HTA) includes the processes and methods used to produce HTA reports with special focus on hospital practice (3) (Table 2). The overarching principle of HB‐HTA is to provide hospital decision‐makers with relevant, comprehensive, objective, and reliable information on the effects and implications of introducing a new HT into the hospital, and the information provided by HB-HTA is analyzed considering the specific context of the hospital where the HT is to be introduced (Table 2). In order to be able to support decision-making in hospitals, HTA should also focus on local infrastructure, prevailing treatment options, patient populations, learning curves, and competing priorities (3).

**TABLE 2:** The dimensions of HB-HTA investigated in the current paper (derived from refs 3 and 4).

Dimension	Item
Clinical	• Safety/risk
	• Efficacy/effectiveness
	• Mortality/survival rates
	• Population to be treated (donors, recipients)
	• Incidence/prevalence of illness
Economic(al)	• Costs
	• Cost-effectiveness, cost utility, cost opportunity
	• Resource(s)
Ethical	• Patient acceptance/comfort
	• Access to novel HT
	• Equity
	• Potential patient harm
Social	• Patient quality of life
	• Pain/discomfort
	• Time in hospital/patient burden
Organizational	• Training
	• Equipment availability/location
	• Resource constraints
Human factors	• Acceptance/acceptability
	• Usability/ease of use

Note. HT, health technology; HB-HTA, hospital-based HTA; HTA, health technology assessment.

Given the paucity of HTA reports on novel HT implemented in LT in general, and on MP in particular, the current paper presents the result of an evidence-based HB-HTA of MP devices for human LT with reference to the European hospital practice and a disease-related group (DRG) reimbursement policy.

## Materials and Methods

In January 2022, we carried out a systematic literature review on MP for adult LT. The literature search explored the Pubmed, CINAHL, Scopus, Embase, and Cochrane databases using a combination of the following MeSH entries with no time limit: #liver transplant (ation), #liver graft, #machine perfusion, #hypothermic machine perfusion, #normothermic machine perfusion, #subnormothermic machine perfusion, #*ex-vivo* machine perfusion, #*ex-situ* machine perfusion, #safety, #complication(s), #risks, #cost(s), #utility, #effectiveness, #efficacy, #outcome(s), #results, #resource(s), #training, #acceptability, #quality of life, #access, #equity, #usability, #population(s), #health technology, #health technology assessment, #hospital(s), and #hospital-based health technology assessment.

The resulting list of references was checked by both investigators, and only papers published in English on clinical application of MP were included. Non-original research works, such as letters to editors, personal points of view, commentaries, and state-of-the-art papers were excluded. Reviews and meta-analyses were considered for data relevant to the current research strategy. The abstracts of all retrieved references were analyzed by the investigators for consistency with the scope of the current research, and if considered relevant the corresponding full papers were included. The articles’ references lists were scanned for evidence of papers not reported in the above databases. In the event of duplicates or manuscripts from the same institution, only the most recent or comprehensive reports were retained. Qualitative assessment of published manuscripts was according to the Currency, Relevance, Authority, Accuracy, Purpose (CRAAP) methodology described elsewhere (5).

Two different clinical settings were included, i.e., DBD and DCD LT using static cold storage (SCS) as the comparator. For both clinical scenarios, the literature was analyzed with the intent to provide information on any of the 6 dimensions and 19 items of the HB-HTA framework as derived by previous works (Table 2) (3, 4). The hospital perspective was that of a national health system (NHS)-based health payer, this being the system in place in Italy and most EU-27 countries, and the corresponding reimbursement policy was that of a DRG-based system. Data on the commercially available MP devices were pooled, since superiority of any HT was beyond the scope of the present analysis. As for any HTA report, the literature review was completed with recommendations and identification of unexplored and underexplored areas and/or items to be investigated in future research. Due to its noninterventional design, no approval by the local ethics committee was necessary as per current Italian regulations.

## Results

**FIGURE 1:**
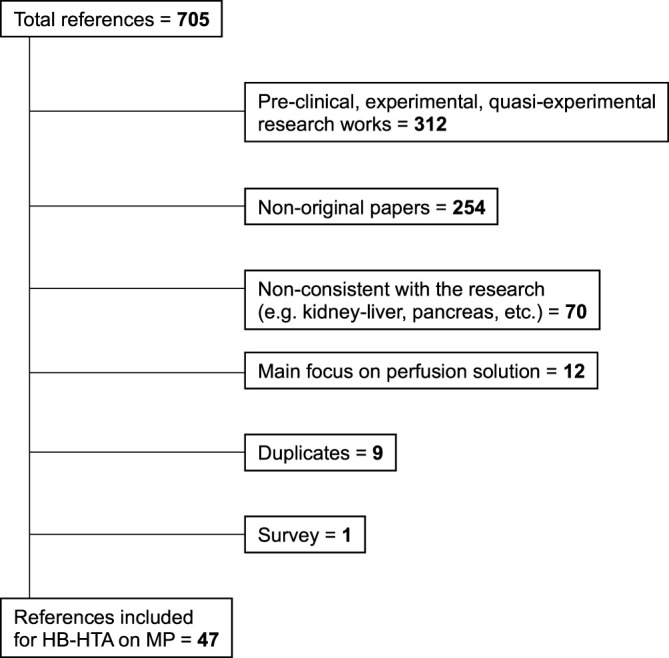
The literature search algorithm. HB-HTA, hospital-based health algorithm; MP, machine perfusion.

Out of 705 references initially retrieved through the databases, 312 (44.2%) papers were excluded being experimental works both in the pre-clinical and clinical setting, 254 (36.0%) were non-original works (letters, expert opinions, state-of-the-art articles, or position papers), 70 (9.9%) were not consistent with the research scope (i.e., combined organ transplantation, pediatric populations, mixed animal and human studies, etc.…), 12 (1.7%) focused mainly on perfusion solutions, 9 (1.3%) were duplicates, and 1 (0.1%) was a survey. Finally, 47 (6.6%) references were retained for current analysis (6–52) (Figure 1). The selected references were published between 2010 and 2022, and all were available as full-length papers.

No previous reference on HTA of MP in human LT was retrieved. The majority of published evidence focused on efficacy/effectiveness of MP in the setting of ECD DBD (6, 8, 10–16, 21–26, 28–38, 40, 41,45, 46, 48, 49, 51) and of type-2 and 3 DCD (7, 8, 13, 16–24, 26, 27, 29–31, 33, 35–48, 50), with most series including both donor populations (Table 3). Information was frequently provided on incidence of biliary complications, which were considered as a surrogate of MP efficacy in most clinical trials together with markers of acute liver injury (9, 17, 30, 31, 33, 34–37, 38, 40, 41). Universal consensus was shared on use of MP in the setting of DCD, especially for type-2 donors, while identification of ECD DBD categories in need of MP was more controversial and yet not entirely agreed upon (10, 16, 28, 32). No consensus on recipient populations to be treated with MP has so far been reported in the international literature, and this choice is usually based on local center allocation policies and regional/national donation rates. Limited information was published on costs and cost-effectiveness/cost utility of MP (13, 21) with original studies originating from Canada (13) and the United Kingdom (21) only. No reference was retrieved on human factors or organizational issues related to the use of MP. Similarly, no information was available on quality of life, access to MP, equity in using MP across diverse patients’ categories, or potential patient harm connected with this novel HT (i.e., competitive models).

**TABLE 3:** Quantitative results of HB-HTA of MP versus SCS (information is presented for items where qualitative information was available).

Dimension	Available information
Clinical	• No increased complication rate (15, 24, 30, 32, 35, 37)
	• Prolonged total graft preservation time (15, 35)
	• 10–50% reduced risk for EAD (7, 17, 24, 30, 36, 37, 41, 47)
	• 7–15% less IRI (7, 17, 28, 32, 39, 42)
	• 7–50% fewer IBC (7, 15, 17, 24, 30, 31, 36, 37, 47)
	• Comparable (22, 24) or improved 1-year graft (30, 47) and patient survival (30, 35)
	• Up to a 50% lower discard rate (31, 35, 43)
Economic(al)	• Increased costs [per-run cost of 18,593.02 $Can (13); per-patient increase of 9,341£ (20)]
	• Theoretically improved cost-effectiveness and cost utility (21)
	• Increased use of economic resources (13, 21)
Ethical	• Anecdotal single reports of MP-related adverse events (37)
Social	• No difference in length of hospital stay (15, 24, 30)

Note. EAD, early allograft dysfunction; HB-HTA, hospital-based health technology assessment; HT, health technology; HTA, health technology assessment; IBC, ischemic biliary complications; IRI, ischemia reperfusion injury; MP, machine perfusion; SCS, static cold storage.

From a quantitative point of view (Table 3), use of MP was associated with definite advantages over SCS. In comparative studies, in light of similar rates of transplant-related (i.e., artery thrombosis) and unrelated (i.e., bleeding) complications (15, 24, 30, 32, 35, 37), MP allowed for prolonged total graft preservation time (15, 35), a 10%–50% reduced risk for EAD (7, 17, 24, 30, 36, 37, 41, 47), 7%–15% less IRI (7, 17, 28, 32, 39, 42), 7–50% fewer ischemic biliary complications (IBC) (7, 15, 17, 24, 30, 31, 36, 37, 47), comparable (22, 24) or improved 1-year graft (30, 47) and patient survival (30, 35), and up to a 50% lower discard rate (31, 35, 43). Hospital stay was not longer for MP patients (15, 24, 30), and technical failures were anecdotal (36). Costs of MP have limitedly been investigated in two studies only (13, 21). One Canadian paper reported a minimum cost per MP run of 18,593.02 $Can, and hypothesized potential cost savings by decreasing night-time salary premiums, complications, and length of hospital stay (13). A study from the United Kingdom focused on costs and cost utility of OrganOx metra™ only, demonstrating higher per-patient costs versus SCS (46,711 versus 37,370£) in light of an anticipated increase in quality of life years (QALY) (10.27 versus 9.09) gained by this novel HT versus SCS (21).

Table 4 illustrates the recommendations derived from the current HB-HTA report.

**TABLE 4:** Key considerations on introduction of MP in the hospital setting based on HB-HTA.

Dimension	Information
Clinical	Available
	• Current MP technology is safe and associated with equal-to- superior graft and patient short-term survival versus SCS
	• Main advantages of MP are a reduced risk for IRI, EAD, and IBC, and a reduced graft discard rate
	• MP facilitates implementation of a DCD LT program, especially for type-2 DCD grafts
	Needed
	• Better identification of ECD DBD grafts to treat with MP
	• Better identification of recipient populations to be treated with MP
	• Long-term data in transplant populations exposed to MP
Economic(al)	Available
	• MP is not economically neutral
	• MP is projected to increase costs of LT in the hospital setting
	• HT advancements are projected to increase MP-related costs in the near future (i.e., graft reconditioning)
	Needed
	• Cost-effective and cost-utility analyses on long-term recipients of MP-facilitated LT
	• Best strategies to neutralize increased costs of MP (i.e., introduction of *ad hoc* DRG, reimbursement of marginal gains achieved from increased proportion of transplants, etc.)
Ethical	Available
	• Limited information is currently available and consists of reports of numerically low MP-related adverse events
	Needed
	• Patient acceptance has to be investigated
	• Strategies to allow for equitable access to MP across LT centers should be identified
	• Potential patient harm from non-implementation of MP-facilitated transplantation should be investigated with simulation models (i.e., competitive risk analysis)
Social	Available
	• None
	Needed
	• Patient quality of life has to be investigated in the setting of MP-facilitated LT
	• Time in hospital/patient burden should be the focus of future studies
Organizational	Available
	• None
	Needed
	• Future studies should focus on staff training and learning curves, equipment availability with regard to comparative analysis of the different commercially available devices, and on the impact of resource constraints (staff and/or financial) on implementation of an MP-facilitated LT program
Human factors	Available
	• None
	Needed
	• As technology evolves, acceptance/acceptability of novel devices and information on usability/ease of use has to be provided

Note. DCD, donation after circulatory death; DRG, disease-related group; EAD. Early allograft dysfunction; ECD, extended criteria donors; HB-HTA, hospital-based HTA; HT, health technology; HTA, health technology assessment; IBC, ischemic biliary complications; LT, liver transplantation; MP, machine perfusion; SCS, static cold storage.

## Discussion

Modern healthcare systems are under pressure and facing challenges that govern their sustainability. One of these challenges is the expansion in technical developments that are fueling innovative and attractive HT to provide answers for unmet medical needs. Innovation is highly rewarding, since it contributes to improved population health status, prolonged life expectancy, and better quality of life. On the other hand, healthcare managers are more accurate in their decisions concerning public expenditure due to the global economic shrinkage. In this scenario, HTA and HB-HTA reports are even more crucial to guide decisions on innovative HT.

MP technology is a recently introduced and expensive intervention whose benefits are under evaluation. With most evidence focusing on patients’ outcomes, limited information is available on the impact of this novel technology on hospitals, healthcare systems, and communities. The paucity of information compliant with standard HTA reports is due to the incredible velocity of research on MP, introduction of this technology at a higher pace than anticipated, and also on the lack of consideration on the part of scientists and clinicians. To the best of our knowledge, this is the first HTA report on MP in LT to be published in the international literature.

Our study confirms that use of MP for LT is safe and associated with frequently improved graft and patient survival for recipients of DCD and ECD DBD transplants, with an associated reduced risk for EAD and ischemic biliary complications. MP seems necessary for implementation of a DCD LT program with special reference to type-2 DCD grafts, due to its striking superiority versus SCS in this setting. But MP also seems to expand use and rescue of ECD grafts, although its implementation in this scenario is frequently driven by regional/national yearly donation rates, proportion of utilized marginal liver grafts, disposition of the waiting list, and single center allocation policies. Future studies should focus on identification of the ideal recipient populations to be treated and on long-term post-transplant survival.

MP is not economically neutral and is projected to increase costs of LT in the hospital setting. Additionally, the evolving scenario of technology advancements is anticipated to increase costs of future MP devices and of those ancillary technologies (i.e., MP-facilitated graft reconditioning) that are currently being explored worldwide. Based on its impact on graft and patient survival, MP-facilitated LT is anticipated to be cost-effective compared to non-transplant best care practices for liver disease patients, but cost-effective and cost-utility analyses require implementation of appropriately powered studies on long-term transplant recipients.

In the economic evaluation of healthcare technologies, costs are usually calculated by multiplying the quantities of resources used per patient by the unit costs of the resources, but economic evaluations for MP technology require alternative approaches to the standard patient-specific modeling by considering all of the following: 1) procedures that generated transplantation versus those that did not generate suitable grafts (i.e., per-run cost); 2) transplantation of liver grafts that would not be otherwise used, and 3) the impact of expanded graft utilization on patients, hospitals, and populations. Especially for NHS-based transplant programs, generating more transplants from use of ECD grafts may increase the economic burden for hospitals (as per the increased number of pre-transplant investigations, surgeries, perioperative care, and post-transplant medical treatment), but these costs should be balanced against those associated with non-transplant care while waiting for a standard quality graft and those derived from loss of transplant-related survival benefit.

To this regard, the choice of the most appropriate costing models and resource-use items is crucial for future analyses, and will require broad consensus across the healthcare professionals involved in LT programs. The decision on which types of cost to include depends on several key factors, including the perspective to be adopted (e.g., hospital managers versus patients versus payors), the form of economic evaluation (e.g., cost-effectiveness versus cost utility versus cost opportunity), the quantitative importance of the type of cost along the entire transplant continuum (i.e., what is the economic burden of MP technology as compared to that related to chronic immunosuppression?), whether the cost can be attributed to the intervention (i.e., can we anticipate reduced cost for treatment of post-transplant ischemic cholangiopathy), and the time horizon of the economic evaluation (perioperative versus early-term versus long-term versus life-long). Collection of detailed data on resource use for all patients may not be necessary, but can be limited to key cost-generating events (normothermic regional perfusion, MP technology, re-transplantation, etc.) where there is economic variation between standard patients and those treated with the novel technology. As clinicians, we are challenged to think through all these methodological issues related to MP technology and build empirical evidence in our future practice.

From the hospital perspective, strategies to neutralize the costs of MP are urgently needed, such as introduction of specific DRG categories, reimbursement of marginal gains retrieved from the increased proportion of transplants, or from out-of-pocket co-pays. Additional avenues for future research should also focus on patient acceptance, on strategies to offer equitable access to MP across different LT centers, and on potential patient harm from non-implementation of MP-facilitated transplantation.

Finally, we advocate future research on staff training and learning curves, on equipment availability with regard to comparative analysis of the different commercially available devices, and on impact of resource constraints (staff and/or financial) on implementation of an MP-facilitated LT program. As technology evolves, acceptance of novel devices and information on usability and ease-of-use from healthcare professionals is also highly needed.

## Abbreviations

CS, cold storage; DBD, donation after brain death; DCD, donation after circulatory death; DRG, disease-related group; EAD, early allograft dysfunction; ECD, extended criteria donors; HB-HTA, hospital-based health technology assessment; HT, health technology; HTA, health technology assessment; IBC, ischemic biliary complications; IRI, ischemia/reperfusion injury; LT, liver transplantation; NHS, national health system; SCS, static cold storage; SD, standard deviation.
